# Ultrasound‐Guided Ovulation Monitoring Versus Home Ovulation Tests and Psychosocial Outcomes in Subfertile Couples: Prospective Cohort Study

**DOI:** 10.1111/1471-0528.70127

**Published:** 2025-12-23

**Authors:** Yueying Zhu, Qi Xi, Zhuo Li, Rulin Dai, Xin Lv, Yang Yu

**Affiliations:** ^1^ Reproductive Medicine Centre and Prenatal Diagnosis Centre The First Hospital of Jilin University Changchun China

**Keywords:** home ovulation tests, ovulation monitoring, sexual function, stress, timed intercourse, ultrasound‐guided

## Abstract

**Objective:**

To assess whether ultrasound‐guided ovulation monitoring, compared to home ovulation tests, increases stress levels or impairs sexual function in subfertile couples.

**Design:**

A prospective cohort study.

**Setting:**

Academic medical centre from November 2023 to October 2024.

**Population:**

A total of 311 subfertile couples are trying to conceive.

**Methods:**

Subfertile couples were allocated to two groups: the ultrasound‐guided ovulation monitoring group and the home ovulation tests group and were surveyed over four menstrual cycles. Stress, anxiety, depression, and sexual function of both partners were investigated through questionnaires.

**Main Outcome Measures:**

Perceived stress scale.

**Results:**

During the four menstrual cycles, neither significant between‐group differences nor group‐by‐cycle interaction effects were observed for stress, anxiety, or depression in either partner (all *p* > 0.05). However, a significant cycle effect was identified over the 4 cycles: anxiety levels increased in women (*p* = 0.037), while both anxiety (*p* < 0.020) and depression (*p* < 0.001) rose significantly in men. Additionally, across the study period, women's Female Sexual Function Index scores (*p* = 0.021) and men's erectile function (*p* < 0.001) showed a significant decline. In terms of pregnancy outcomes, the ultrasound‐guided group achieved a pregnancy rate twice that of the test group (adjusted OR = 2.12, 95% CI 1.32–3.39, *p* = 0.002).

**Conclusion:**

Ultrasound‐guided ovulation monitoring shortens time to conception, with no evidence of increased emotional distress or impaired sexual function.

## 1 Introduction

Infertility is a global health issue affecting approximately 10%–15% of couples, with significant psychological, social and economic costs [1, 2]. To achieve conception quickly, couples typically choose to have timed intercourse during the fertile window, the period with the highest likelihood of pregnancy [3]. However, a substantial proportion of women are unable to accurately identify their fertile window [4]. This is primarily because they are unaware of their ovulation timing or hold misconceptions about it [5, 6, 7]. Even women with regular ovulatory cycles show large cycle‐length variability within and between themselves [8].

Home ovulation tests provide a straightforward and non‐invasive method for pinpointing the ideal moment for intercourse [9]. However, premature ovulation relative to the expected LH‐triggered date occurs in nearly 10% of cycles, while late ovulation is observed in 23% of cycles [10]. Direct follicular monitoring through transvaginal ultrasound in a hospital setting can accurately determine the timing of ovulation, and it is known for higher pregnancy rates compared with home ovulation tests [11, 12]. However, due to its invasive nature, the economic costs and the time burden of repeated hospital appointments, ultrasound‐guided monitoring is primarily utilised in assisted reproductive technology (ART) to precisely measure the fertile window [13, 14]. Choosing between home ovulation tests and hospital ultrasound‐guided monitoring depends on couples' needs and clinicians' awareness of each method's impact on stress and sexual function. The relationship between stress and fertility is intricate and reciprocal: stress can potentially diminish fertility, while fertility challenges can intensify stress [15]. Stress can affect ovulation and sperm production through its impact on endocrine regulation, while timed intercourse aimed at conception can reduce sexual satisfaction and increase stress [16, 17].

Studies show no difference in stress or sexual function between home test users and non‐trackers. However, as the duration of infertility extends, couples experience significant negative impacts on their stress levels and sexual function [18, 19]. Indeed, failure to become pregnant is likely to be the primary source of stress [20]. Ultrasound‐guided monitoring offers the advantage of high accuracy and pregnancy rates, but there are concerns about its potential adverse effects on stress and sexual function. Regrettably, to date, there has been a lack of relevant research.

Therefore, we conducted a prospective cohort study comparing the two methods of follicular monitoring, aiming to determine whether ultrasound‐guided ovulation monitoring has adverse effects on stress and sexual function in subfertile couples compared to home ovulation tests.

## 2 Methods

This was a prospective cohort study. The protocol was approved by the medical ethics committee of the First Hospital of Jilin University (approval number: 23 K219‐001) and was registered on ClinicalTrials.gov prior to its initiation (identifier: NCT06127875). All participants provided written informed consent, and the study was conducted in accordance with the Helsinki Declaration.

### 2.1 Participants

Participants were recruited from the reproductive outpatient clinic of the reproductive medicine centre at The First Hospital of Jilin University between November 2023 and October 2024. The following inclusion criteria were used and presented to potential participants before informed consent: (i) Couples with regular sexual activity, no contraception for over 6 months, and had not previously used home ovulation tests or ultrasound‐guided ovulation monitoring; (ii) female participants aged between 20 and 40 years, who had regular menstrual bleeds and wished to conceive. Male participants aged between 22 and 45 years; (iii) the couple would utilise home ovulation tests or ultrasound‐guided ovulation monitoring to facilitate conception. Exclusion criteria: (i) Couples with contraindications to pregnancy; (ii) male participants diagnosed with severe oligospermia or azoospermia (sperm concentration was less than 1 × 10^6^/ml); (iii) anyone with a history of depression or anxiety. Participants were divided into two groups based on the ovulation monitoring method they chose: the ultrasound‐guided ovulation monitoring group (ultrasound group) and the home ovulation tests group (test group). Eligible couples who agreed to participate provided informed consent under the guidance of a physician. The participants chose the method of ovulation monitoring by themselves.

### 2.2 Study Design

Questionnaire assessments were made before (T0), after two ovulation monitoring cycles (T1) and after four ovulation monitoring cycles (T2) following enrolment. Follow‐ups were conducted via WeChat, a popular instant messaging service, with up to three reminders and the option to unsubscribe at any time. Participants were followed from study entry until pregnancy or the end of the follow‐up period. Once pregnancy was confirmed, questionnaire administration for that cycle is discontinued. Monitoring protocols were as follows:

Ultrasound group: Transvaginal ultrasound monitoring was initiated on cycle days 10–12, with scans repeated every 1–3 days based on follicular diameter. Once a dominant follicle reached ≥ 18 mm, the couple was advised to have intercourse on that day and again 24–48 h later. A total of approximately 3–4 clinic visits were required for this monitoring cycle.

Test group: All participants were instructed to use qualitative strips (colloidal‐gold method). Starting on cycle day 10, women performed daily urine luteinising hormone (LH) testing at home. Couples were instructed to have intercourse on the day an LH peak was detected and again on the day the test strip returned to negative. Couples assigned to home ovulation tests received instructions on the methods to be used after enrolment. When the first positive LH result was obtained, couples could send a photo of the test strip to the physician for online consultation and further instructions.

### 2.3 Clinical, Demographic and Background Information

Detailed demographic and clinical histories were obtained for all couples. Demographic data included age, education level, and body mass index (BMI). Clinical data included the reasons for medical consultation, duration of cohabitation, infertility history, pregnancy and delivery history of the female, menstrual history, as well as the male's sexual function and sperm parameters.

### 2.4 Measurement Variables

The primary outcome was perceived stress, with anxiety, depression, sexual function and pregnancy rate serving as secondary endpoints.

The assessment of stress levels was conducted using the Perceived Stress Scale (PSS) [21]. Participants were required to rate their stress experiences on a five‐point Likert scale, with 1 representing ‘never’ and 5 indicating ‘very often’ across a total of 14 items. The total scores were indicative of the level of stress, with higher values reflecting greater stress levels.

The Hospital Anxiety and Depression Scale (HADS) was used to assess anxiety and depression symptoms [22]. This scale encompasses two domains, each comprising seven items that are scored on a scale from 0 to 3, culminating in a total score that ranges from 0 to 21.

Female sexual function was assessed using the Female Sexual Function Index (FSFI) [23], a 19‐item questionnaire that evaluates six dimensions of sexual function: desire, arousal, lubrication, orgasm, satisfaction, and pain. Each item is scored on a five‐point Likert scale from 0 (or 1) to 5, with lower scores indicating reduced sexual function. For male participants, sexual erectile function was evaluated using the International Index of Erectile Function (IIEF) [24], which has five items focusing on erectile function and sexual satisfaction. Each item is scored on a five‐point Likert scale from 1 to 5, with higher scores indicating better sexual functioning. Additionally, the Premature Ejaculation Diagnostic Tool (PEDT) was used to diagnose premature ejaculation in males [25]. The PEDT is a concise self‐report questionnaire with five items in total, scored on a scale from 0 to 4, resulting in a total score that ranges from 0 to 20, where lower scores indicate better sexual function.

### 2.5 Statistical Analysis

Based on the research by Tiplady et al., to achieve 80% statistical power at a significance level of 0.05 and to detect a between‐group difference of 2.02 units on the primary outcomes with a medium effect size (SD = 5.0) using the same stress assessment, a minimum of 97 participants per group is required [19]. Considering that 21%–27% of participants might withdraw from the study due to ineligibility or pregnancy [26], a minimum sample size of 123 couples per group is required to ensure statistical adequacy. To account for potential attrition, we aimed to recruit 155 couples per group.

To minimise baseline confounding, we performed 1:1 propensity‐score matching (PSM) (calliper = 0.1 SD of the logit) on female age, male age, female BMI, education, infertility duration, previous pregnancy, live births, pregnancy losses, and baseline PSS scores; after matching, all standardised mean differences fell < 0.10. Variables with qualitative characteristics were described as frequencies, and variables with quantitative characteristics were described as mean ± standard deviation (SD). Comparisons between groups were performed with Student's t‐test for continuous parametric variables and with χ^2^ or Fisher's exact test for categorical variables. Psychosocial and sexual function outcomes were analysed with linear mixed‐effects models (LMMs), with fixed effects for group, cycle, their interaction and all covariates retained in the PSM. Time to pregnancy was analysed with Cox proportional hazards regression in the matched cohort, adjusting for the same covariates. Statistical significance was determined by a two‐tailed value of *p* < 0.05. Statistical analyses were processed using IBM SPSS Statistics for Windows, version 27.0 (Armonk, NY: IBM Corp.) software package.

## 3 Results

### 3.1 Recruitment and Response

**FIGURE 1:**
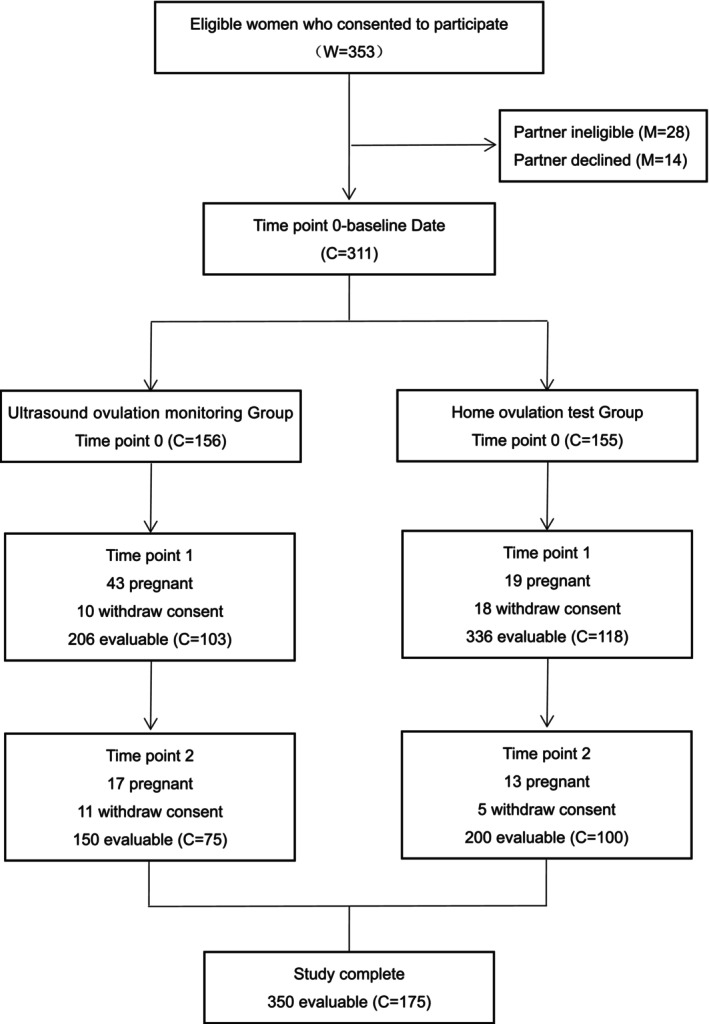
Flowchart summarising the numbers of participants at each time point during the study. W, women; M, men; C, couple.

Figure 1 summarises participant flow. Among 353 women screened, 28 couples were excluded because of severe semen abnormalities and 14 refused participation. The remaining 311 couples completed the baseline questionnaire and were allocated to the ultrasound group (*n* = 156) or test group (*n* = 155). During follow‐up, 92 women ceased further data collection due to pregnancy, and another 44 couples withdrew voluntarily or were lost to follow‐up. A total of 175 couples completed all three rounds of questionnaires.

### 3.2 Demographic and Clinical Characteristics of the Participants

Table 1 depicts couples' characteristics before and after 1:1 PSM. Pre‐matching, the ultrasound group exhibited a longer mean infertility duration (16.1 ± 16.2 vs 10.8 ± 7.9 months, *p* < 0.001) and a higher infertility rate (47.4% vs 29.0%, *p* < 0.001). Age, BMI, education, previous pregnancy, live births, pregnancy losses, and baseline PSS were comparable.

**TABLE 1:** Participants' demographic and clinical characteristics before and after propensity‐score matching.

	Before matching			After matching (*)			
	Ultrasound group	Test group	*p*	Ultrasound group	Test group	Total	*p*
	(C = 156)	(C = 155)		(C = 127)	(C = 127)	(C = 254)	
Age (year)							
Female	30.7 ± 3.4	30.7 ± 3.4	0.863	30.9 ± 3.4	30.8 ± 3.4	30.8 ± 3.4	0.739
Male	32.2 ± 3.4	31.6 ± 3.8	0.115	32.1 ± 3.4	31.8 ± 3.8	31.9 ± 3.6	0.541
Female BMI (kg/m^2^)	23.4 ± 3.8	22.9 ± 4.0	0.215	23.1 ± 3.7	22.8 ± 3.9	23.0 ± 3.8	0.556
Female Education level, *n* (%)			0.347				0.860
No higher than university	28(18.0)	19(12.3)		16(12.6)	18(14.2)	34(13.4)	
University	105(67.3)	109(70.3)		91(71.7)	87(68.5)	178(70.1)	
University above	23(14.7)	27(17.4)		20(15.7)	22(17.3)	42(16.5)	
Trying to conceive (months)	16.1 ± 16.2	10.8 ± 7.9	**< 0.001**	11.1 ± 8.2	11.6 ± 8.4	11.4 ± 8.3	0.684
Infertility (*n*, %)			**< 0.001**				0.600
Yes	74(47.4)	45(29.0)		47(37.0)	43(33.9)	90(35.4)	
No	82(52.6)	110(71.0)		80(63.0)	84(66.1)	164(64.6)	
Previous pregnancy *n*(%)			0.246				0.659
Yes	42(26.9)	33(21.3)		32(25.2)	29(22.8)	61(24.0)	
No	114(73.1)	122(78.7)		95(74.8)	98(77.2)	193(76.0)	
Live births	8(5.1)	4(2.6)	0.378	4(3.1)	4(3.1)	8(3.1)	1.000
Pregnancy losses	26(16.7)	25(16.1)	0.898	23(18.1)	21(16.5)	44(17.3)	0.740
Baseline PSS	37.4 ± 8.1	37.0 ± 7.3	0.666	37.1 ± 7.8	37.1 ± 7.5	37.1 ± 7.6	0.980

*Note: Values* in bold are *p* < 0.05.

Abbreviations: BMI: body mass index; C, couple.

* The matching covariates included female age, male age, female BMI, education, infertility duration, previous pregnancy, live births, pregnancy losses, and baseline PSS scores.

Post‐matching, 254 couples (127 per arm) remained. Standardised mean differences were < 0.10 for all covariates, confirming adequate balance. The matched sample had a mean female age 30.8 ± 3.4 years, a mean male age 31.9 ± 3.6 years, and a mean time of 11.4 ± 8.3 months trying to conceive; 35.4% had an infertility diagnosis. Obstetric history was reported by 24.0% of couples, 3.1% had a previous live birth, and 17.3% had experienced pregnancy loss. Over 85% of women held a university degree or higher. Mean baseline PSS was 37.1 ± 7.6. No significant between‐group differences were observed for any variable.

### 3.3 Psychosocial Outcomes

Table 2 presents the means ± SD for psychosocial outcomes for both partners at baseline (T0), after 2 cycles (T1) and after 4 cycles (T2), with significance tests derived from LMMs. No between‐group differences (ultrasound vs. test) were observed at any assessment for either partner, and the group‐by‐cycle interaction was non‐significant for psychosocial outcomes. Cycle number did not have a significant effect on stress in either partner (PSS: *p* = 0.778 and *p* = 0.866, respectively). Anxiety increased significantly in women (*p* = 0.037), whereas depression showed no significant change (*p* = 0.168). In men, both anxiety (*p* = 0.020) and depression (*p* < 0.001) showed significant increases over time.

**TABLE 2:** Descriptive statistics (mean ± SD) for psychosocial outcomes and linear mixed‐effects significance of effects for group, cycle and group‐by‐cycle interaction (*N* = 254).

		Ultrasound group (*n* = 127)	Test group (*n* = 127)	Coefficients	SE	95% CI	*p*
Female PSS							
Intercept				46.43	6.7	33.32, 59.54	< 0.001
Cycle	Baseline (T0)	37.1 ± 7.8	37.1 ± 7.5	Reference			
	2 cycles (T1)	37.0 ± 7.6	36.2 ± 7.5	−0.48	0.66	−1.77, 0.81	0.465
	4 cycles (T2)	37.0 ± 7.6	37.2 ± 7.4	0.20	0.70	−1.18, 1.57	0.778
Group				0.18	0.96	−1.70, 2.07	0.461
Group*Cycle				—	—	—	0.568
Depression							
Intercept				6.60	3.02	0.65, 12.55	0.030
Cycle	Baseline (T0)	5.5 ± 3.4	5.8 ± 3.4	Reference			
	2 cycles (T1)	5.8 ± 3.3	5.7 ± 3.4	−0.06	0.29	−0.62, 0.51	0.847
	4 cycles (T2)	5.8 ± 3.6	6.5 ± 3.4	0.42	0.31	−0.18, 1.03	0.168
Group				−0.24	0.43	−1.09, 0.61	0.954
Group*Cycle				—	—	—	0.325
Anxiety							
Intercept				9.78	3.08	3.72, 15.84	0.002
Cycle	Baseline (T0)	5.8 ± 3.6	5.8 ± 3.6	Reference			
	2 cycles (T1)	6.0 ± 3.3	5.7 ± 3.8	0.13	0.35	−0.56, 0.82	0.708
	4 cycles (T2)	6.2 ± 3.6	6.4 ± 4.0	0.78	0.37	0.05, 1.51	**0.037**
Group				0.10	0.46	−0.79, 1.00	0.777
Group*Cycle				—	—	—	0.707
Male PSS							
Intercept				43.79	6.49	31.02, 56.57	< 0.001
Cycle	Baseline (T0)	34.3 ± 7.3	35.8 ± 7.6	Reference			
	2 cycles (T1)	35.2 ± 7.0	36.2 ± 8.2	0.30—	0.71	−1.09, 1.69	0.676
	4 cycles (T2)	35.0 ± 7.4	36.2 ± 7.7	0.13	0.75	−1.35, 1,61	0.866
Group				0.34	1.19	−1.99, 2.67	0.415
Group*Cycle				—	—	—	0.617
Depression							
Intercept				7.22	2.81	1.69, 12.75	0.011
Cycle	Baseline (T0)	5.6 ± 3.3	5.4 ± 2.9	Reference			
	2 cycles (T1)	5.7 ± 3.5	6.5 ± 3.2	1.11	0.32	0.48, 1.73	**< 0.001**
	4 cycles (T2)	5.9 ± 3.6	6.8 ± 3.5	1.38	0.34	0.71, 2.05	**< 0.001**
Group				0.22	0.42	−0.60, 1.04	0.355
Group*Cycle				—	—	—	0.118
Anxiety							< 0.001
Intercept				7.97	2.85	2.35, 13.58	0.006
Cycle	Baseline (T0)	4.8 ± 3.3	5.4 ± 3.0	Reference			
	2 cycles (T1)	5.4 ± 3.6	5.7 ± 3.5	0.22	0.35	−0.47, 0.90	0.538
	4 cycles (T2)	5.7 ± 3.7	6.3 ± 4.1	0.87	0.37	0.14, 1.60	**0.020**
Group				−0.48	0.43	−1.33, 0.37	0.466
Group*Cycle				—	—	—	0.649

*Note:* Values in bold are *p* < 0.05.

Abbreviations: CI, confidence interval; PSS, Perceived Stress Scale; SE, standard error.

### 3.4 Sexual Functioning Outcomes

**FIGURE 2:**
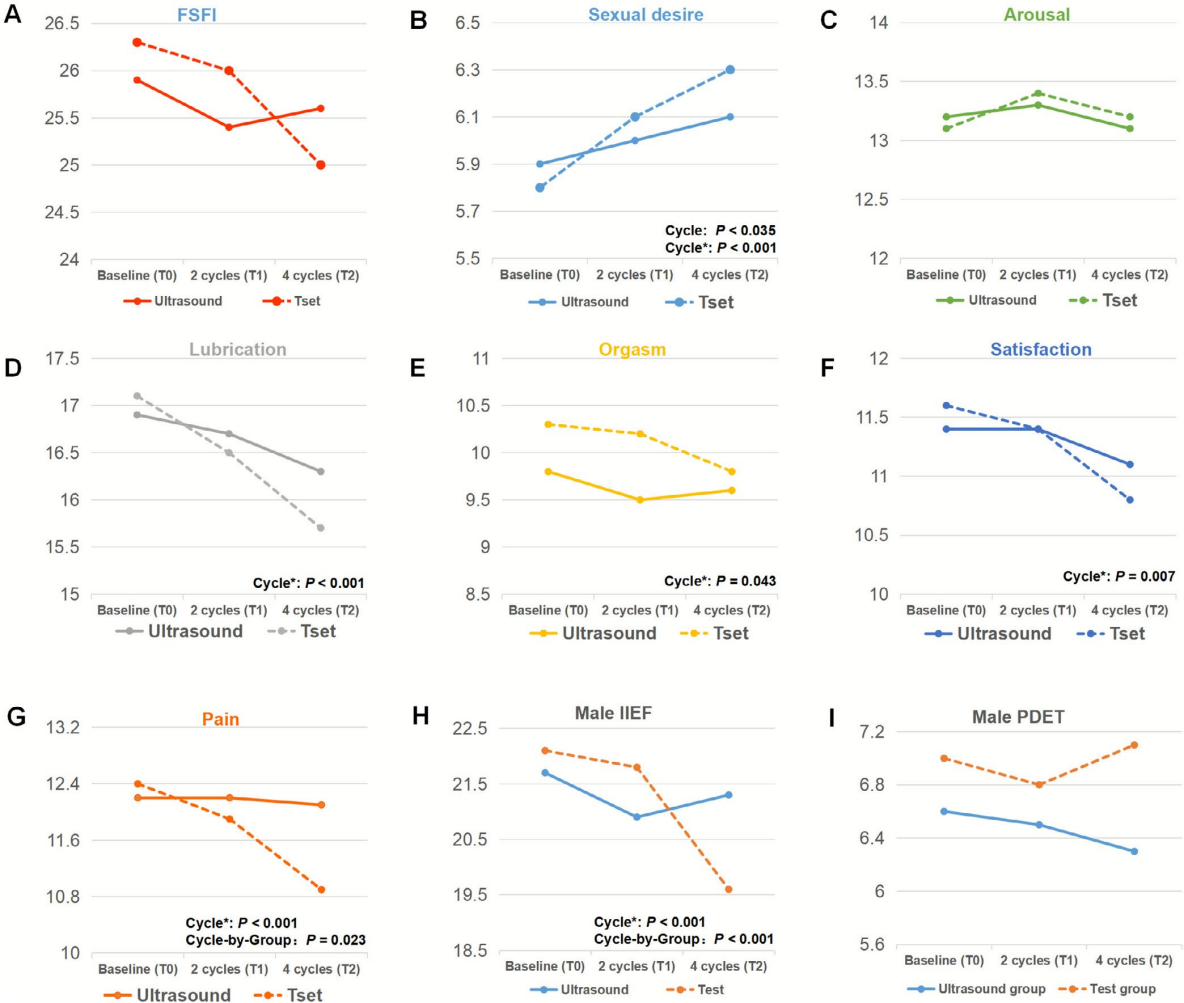
The levels of various dimensions of female sexual function, including FSFI (A), sexual desire (B), arousal (C), lubrication (D), orgasm (E), satisfaction (F), pain (G), male IIEF (H), and male PEDT (I) in the ultrasound group (—) and the test group (−‐‐) before (baseline) and after intervention at 2 cycles and 4 cycles follow‐up. FSFI, Female Sexual Function Index; IIEF, International Index of Erectile Function; PDET, Premature Ejaculation Diagnostic Tool.

Female sexual function reports and results are presented in Figure 2 and Table S1. For women, the total score of the FSFI showed no significant main effects of group or group‐by‐cycle interaction. However, as the number of cycles increased, female FSFI (T2 vs. T0, *p* = 0.021), lubrication (T2 vs T0, *p* < 0.001), orgasm (T2 vs T0, *p* = 0.043), satisfaction (T2 vs T0, *p* = 0.007), and pain scores (T2 vs T0, *p* < 0.001) significantly decreased, whereas desire levels improved (T1 vs T0, *p* = 0.035; T2 vs T0, *p* < 0.001). Pain also demonstrated a significant group‐by‐cycle interaction effect (*p* = 0.023), indicating that the test group experienced a significant increase in pain across cycles, whereas the ultrasound group did not.

For men, the IIEF analyses revealed both a significant group‐by‐cycle interaction (*p* < 0.001) and a significant main effect of cycle (*p* < 0.001) for erectile function. Although erectile scores declined markedly across cycles (T2 vs T0, *p* < 0.001), this decline was confined to the test group (T2 vs T0, *p* < 0.001). Conversely, PEDT results showed no significant group, cycle, or interaction effects on ejaculatory function.

### 3.5 Pregnancy

Pregnancy status was reported for 78 out of 254 women (51 cases [40.2%] in the ultrasound group and 27 cases [21.3%] in the test group). Fifty two pregnancy tests were positive within 2 cycles after baseline. Four cycles later, 26 new pregnancies had occurred. After adjusting for female and male age, previous pregnancy history, live birth history, pregnancy losses, infertility status, duration of attempted conception, female education, and female BMI, the association between monitoring method and pregnancy outcome remained essentially unchanged (Table S2). The odds of achieving pregnancy in the ultrasound group were 2 times those in the test group (adjusted OR = 2.12, 95% CI 1.32–3.39, *p* = 0.002).

## 4 Discussion

### 4.1 Main Findings

This is the first study to compare stress levels and sexual function in subfertile couples undergoing ultrasound‐guided ovulation monitoring versus home ovulation tests. Our study found no evidence of significant differences in stress, anxiety, depression levels between the two groups, although both treatment approaches were correlated with a rise in anxiety and depression. Regarding female sexual function, while both methods induced changes in the FSFI total score and several dimensions as the number of cycles increased, no significant differences were observed between the two groups. Surprisingly, there was a significant decline in male erectile function in the test group as the number of cycles increased, which was significantly different from that in the ultrasound group.

Timed intercourse is considered an effective method for couples to achieve pregnancy quickly [27]. Home ovulation tests are characterised by ease of use and low cost, making them the most commonly employed method among couples seeking natural fertility [28]. Unfortunately, only 13% of these couples correctly identify their fertile window [29]. Ultrasound‐guided monitoring, which is more time‐consuming and costly but offers greater accuracy, is chosen primarily for the following reasons: (i) Couples do not have an absolute indication for ART [30]; (ii) those who have unsuccessfully used home ovulation tests multiple times; (iii) couples who have been trying to conceive for an extended period, are of advanced age, or experience significant fertility‐related stress and have an urgent desire to become pregnant [31]; (iv) couples requiring ovulation induction treatment. For these situations, undergoing 3 cycles of ultrasound‐guided ovulation monitoring to guide timed intercourse is an acceptable option. Couples who have not conceived after 4 or 5 cycles of ultrasound monitoring may consider alternative treatment strategies, as the pregnancy rate is unlikely to increase significantly with further cycles [31]. The choice of this study to investigate 4 cycles was realistic and aligned with clinical practice.

In China, the focus on women's and children's health has led to the establishment of specialised maternal and child health hospitals across counties and cities. As a routine basic examination, ultrasound monitoring is widely accessible in county‐level hospitals and community healthcare centres. With its low economic and time costs, it has become a common choice among infertile couples. However, across different countries and regions, varying healthcare systems and personal circumstances mean that both economic and time costs should be carefully weighed when choosing a monitoring strategy, with decisions tailored to local contexts.

### 4.2 Psychological

However, there is a lack of research on the impact of ultrasound monitoring on psychological and sexual function. In this study, there was no difference in stress levels between the two groups compared to the baseline, and there were no intergroup differences in changes over time. When comparing home ovulation tests with a non‐monitoring group, no significant difference in stress between the two groups was found, nor was there evidence of changes over time [19]. This suggests that focusing on the fertile window does not increase stress in couples, and this study further indicates that ultrasound monitoring does not lead to increased stress. We believe our study is very important because it provides new evidence for the choice of ovulation monitoring protocols. Our results also showed that depression and anxiety levels significantly increased in both groups, with no intergroup differences noted. There is evidence that patients with a history of infertility treatment failure and pregnancy loss experience exacerbated depressive and anxiety symptoms in subsequent pregnancies [32, 33]. However, several studies have examined the relationship between psychological factors and the outcomes of ART, and the results indicate a lack of correlation between such emotional distress and pregnancy outcomes [34, 35].

### 4.3 Sexual Function

Anxiety and depression in infertile couples are known to be correlated with a decline in sexual function [36]. Among women, no significant intergroup differences were observed in various dimensions of sexual function. However, as the number of cycles increased, changes were noted in most domains. Early in treatment, women consciously increased sexual activity, resulting in higher sexual desire than usual. However, this increase in sexual desire, driven by the goal of conception, can lead to some decline in sexual function and adverse experiences [37]. Previous studies have shown a comprehensive decline in female sexual function, which aligns with the findings of this study, albeit with some differences [38].

In male erectile function assessment, the test group showed a noticeable decline over time, whereas the ultrasound group showed no decline in erectile function. Some studies suggest that men's shift from recreational to procreative sex may initially be pleasant, but this does not mean sexual function declines from the start [39]. Ultrasound monitoring, with high follicular prediction accuracy, often guides doctors to recommend intercourse closer to ovulation. In contrast, the test group men may have earlier erectile function decline. This difference may be related to their anxiety about the timing of ovulation and the procreative purpose of sexual intercourse.

### 4.4 Pregnancy Rate

The pregnancy outcomes of this study indicate that ultrasound monitoring is significantly more effective in aiding women to conceive successfully than home ovulation tests. The ultrasound‐guided group achieved a pregnancy rate twice that of the test group. It is evident that the ultrasound monitoring offers distinct advantages in terms of pregnancy outcomes.

### 4.5 Strengths and Limitations

The strength of this study is attributed to its prospective design, which allows for timely and accurate assessment of changes in psychological status and sexual function among subfertile couples during the monitoring cycle. Moreover, we simultaneously evaluated male psychological status and sexual function. This dimension has been under‐represented in previous research and provides essential evidence for comprehensive clinical assessment and couple‐based intervention.

However, it is important to acknowledge the limitations inherent in our study's design and execution: First, as an observational, non‐randomised controlled study, blinding was not feasible; self‐selected monitoring methods and pregnancy‐related dropouts may have introduced selection and attrition biases, and residual confounding cannot be completely ruled out despite the use of PSM and LMMs. Second, single‐centre recruitment produced a fairly homogeneous sample, so results may not apply to wider, more diverse populations. Third, the brand of LH urine test strips was not standardised, which could have introduced heterogeneity. Nevertheless, all participants were issued qualitative colloidal‐gold tests after enrolment and had their first positive result photo‐verified by physicians, largely removing brand‐related variability. Fourth, we did not measure the economic or time burden of monitoring, which could act as confounders affecting couples' psychological states.

Future research should use multi‐centre, prospective randomised controlled studies and include measures of time and financial costs to refine the outcome set. Identifying which infertile subgroups benefit most from ultrasound monitoring will also help avoid unnecessary burdens.

## 5 Conclusions

In summary, our study found no evidence of significant differences in stress, anxiety, depression levels, or female sexual function between couples using home ovulation tests and those undergoing ultrasound‐guided ovulation monitoring. Ultrasound‐guided monitoring has a minimal impact on male erectile function. Employing ultrasound‐guided monitoring was associated with a shorter time to conception, and we found no evidence of a difference in emotional distress or sexual function between the two groups. It is crucial to provide personalised management for each couple attempting to conceive, selecting the most optimal plan to meet their specific needs and objectives.

## Author Contributions

Y.Z. and Y.Y. designed and conceived the study; Q.X. and Y.Z. drafted the article; X.L. performed the statistical analysis; Data collected by Z.L. and Q.X.; RL.D. supervised the project; Y.Y. made critical revisions to the manuscript. All authors revised and commented on the article and approved the final version before submission.

## Funding

This work was supported by grants from the Science and Technology Department of Jilin Province, China and Science and Technology Development Plan Project of Jilin Province, China (20210101354JC).

## Ethics Statement

The protocol was approved by the medical ethics committee of the First Hospital of Jilin University (approval number: 23K219‐001) and was registered on ClinicalTrials.gov prior to its initiation (identifier: NCT06127875). Date of registration: 11 August 2023. Date of enrolment of the first subject: 1 November 2023. All participants provided written informed consent, and the study was conducted in accordance with the Helsinki Declaration.

## Conflicts of Interest

The authors declare no conflicts of interest.

## Supporting information


**Table S1:** Descriptive statistics (mean ± SD) for sexual function outcomes and linear mixed‐effects significance of effects for group, cycle and group‐by‐cycle interaction (*N* = 254).
**Table S2:** Cox regression analysis of time‐to‐pregnancy between groups (N = 254).

## Data Availability

The data that support the findings of this study are available from the corresponding author upon reasonable request.
