# Supplementary material for: Ultrasound‐Guided Ovulation Monitoring Versus Home Ovulation Tests and Psychosocial Outcomes in Subfertile Couples: Prospective Cohort Study
Source: BJOG. 2025 Dec 23;133(5):996–1005. doi: 10.1111/1471-0528.70127 (PMC12972848; doi:10.1111/1471-0528.70127)
Supplement: Supplementary file 1 — Table S1: Descriptive statistics (mean ± SD) for sexual function outcomes and linear mixed‐effects significance of effects for group, cycle and group‐by‐cycle interaction (N = 254). Table S2: Cox regression analysis of time‐to‐pregnancy between groups (N = 254). [file BJO-133-996-s001.docx]

| **Table S1** Descriptive statistics (mean ± SD) for sexual function outcomes and linear mixed-effects significance of effects for group, cycle and group-by-cycle interaction (N = 254). | | | | | | | |
| --- | --- | --- | --- | --- | --- | --- | --- |
|  | | Ultrasound group  (n = 127) | Test group  (n = 127) | Coefficients | SE | 95%CI | P |
| **Female FSFI** | |  |  |  |  |  |  |
| Intercept | |  |  | 24.55 | 4.08 | 16.51, 32.60 | <0.001 |
| Cycle | Baseline (T0) | 25.9±4.3 | 26.3±4.2 | Reference | | | |
|  | 2 cycles (T1) | 25.4±5.6 | 26.0±4.8 | -0.27 | 0.42 | -1.10, 0.56 | 0.519 |
|  | 4 cycles (T2) | 25.6±4.6 | 25.0±5.4 | -1.04 | 0.45 | -1.93, -0.16 | **0.021** |
| Group | |  |  | -0.38 | 0.59 | -1.54, 0.79 | 0.566 |
| Group*Cycle | |  |  | - | - | - | 0.228 |
| **Sexual desire** | |  |  |  |  |  |  |
| Intercept | |  |  | 6.96 | 1.26 | 4.48, 9.45 | <0.001 |
| Cycle | Baseline (T0) | 5.9±1.4 | 5.8±1.5 | Reference | | | |
|  | 2 cycles (T1) | 6.0±1.5 | 6.1±1.5 | 0.31 | 0.14 | 0.02, 0.59 | **0.035** |
|  | 4 cycles (T2) | 6.1±1.4 | 6.3±1.7 | 0.59 | 0.15 | 0.28, 0.89 | **<0.001** |
| Group | |  |  | 0.10 | 0.19 | -0.27, 0.47 | 0.614 |
| Group*Cycle | |  |  | - | - | - | 0.322 |
| **Arousal** | |  |  |  |  |  |  |
| Intercept | |  |  | 13.15 | 3.10 | 7.04, 19.3 | <0.001 |
| Cycle | Baseline (T0) | 13.2±3.3 | 13.1±3.4 | Reference | | | |
|  | 2 cycles (T1) | 13.3±3.8 | 13.4±3.8 | 0.27 | 0.33 | -0.38, 0.91 | 0.415 |
|  | 4 cycles (T2) | 13.1±3.9 | 13.2±4.0 | 0.20 | 0.35 | -0.49, 0.88 | 0.574 |
| Group | |  |  | -0.00 | 0.45 | -0.89, 0.89 | 0.679 |
| Group*Cycle | |  |  | - | - | - | 0.799 |
| **Lubrication** | |  |  |  |  |  |  |
| Intercept | |  |  | 12.78 | 2.68 | 7.50, 18.06 | <0.001 |
| Cycle | Baseline (T0) | 16.9±3.0 | 17.1±2.6 | Reference | | | |
|  | 2 cycle (T1) | 16.7±3.5 | 16.5±3.4 | -0.58 | 0.35 | -1.26, 0.10 | 0.096 |
|  | 4 cycle (T2) | 16.3±3.6 | 15.7±4.3 | -1.34 | 0.37 | -2.07, -0.62 | **<0.001** |
| Group | |  |  | -0.21 | 0.41 | -1.02, 0.60 | 0.860 |
| Group*Cycle | |  |  | - | - | - | 0.485 |
| **Orgasm** | |  |  |  |  |  |  |
| Intercept | |  |  | 6.14 | 2.59 | 1.03, 11.25 | 0.019 |
| Cycle | Baseline (T0) | 9.8±2.8 | 10.3±2.7 | Reference | | | |
|  | 2 cycle (T1) | 9.5±3.1 | 10.2±2.8 | -0.21 | 0.26 | -0.72, 0.30 | 0.415 |
|  | 4 cycle (T2) | 9.6±3.1 | 9.8±3.5 | -0.56 | 0.27 | -1.10, -0.02 | **0.043** |
| Group | |  |  | -0.58 | 0.37 | -1.31,0.16 | 0.228 |
| Group*Cycle | |  |  | - | - | - | 0.165 |
| **Satisfaction** | |  |  |  |  |  |  |
| Intercept | |  |  | 12.38 | 2.02 | 8.40, 16.36 | <0.001 |
| Cycle | Baseline (T0) | 11.4±2.3 | 11.6±2.4 | Reference | | | |
|  | 2 cycle (T1) | 11.4±2.4 | 11.4±2.5 | -0.26 | 0.21 | -0.66,0.14 | 0.206 |
|  | 4 cycle (T2) | 11.1±2.4 | 10.8±2.3 | -0.60 | 0.22 | -1.03, -0.17 | **0.007** |
| Group | |  |  | -0.24 | 0.29 | -0.81, 0.34 | 0.671 |
| Group*Cycle | |  |  | - | - | - | 0.707 |
| **Pain** | |  |  |  |  |  |  |
| Intercept | |  |  | 10.07 | 2.20 | 5.74, 14.41 | <0.001 |
| Cycle | Baseline (T0) | 12.2±2.5 | 12.4±2.4 | Reference | | | |
|  | 2 cycle (T1) | 12.2±3.0 | 11.9±2.9 | -0.47 | 0.31 | -1.07, 0.14 | 0.133 |
|  | 4 cycle (T2) | 12.1±2.5 | 10.9±3.5^#^ | -1.50 | 0.33 | -2.15, -0.85 | **<0.001** |
| Group | |  |  | -0.18 | 0.35 | 5.74, 0.50 | 0.174 |
| Group*Cycle | |  |  | - | - | - | **0.023** |
| **Male IIEF** | |  |  |  |  |  |  |
| Intercept | |  |  | 22.79 | 2.72 | 17.43, 28.14 | <0.001 |
| Cycle | Baseline (T0) | 21.7±2.7 | 22.1±2.6 | Reference | | | |
|  | 2 cycle (T1) | 20.9±3.9 | 21.8±2.9 | -0.28 | 0.38 | -1.02, 0.47 | 0.466 |
|  | 4 cycle (T2) | 21.3±4.1 | 19.6±5.0^#^ | -2.44 | 0.40 | -3.24, -1.65 | **<0.001** |
| Group | |  |  | -0.43 | 0.43 | -1.27,0.41 | 0.677 |
| Group*Cycle | |  |  | - | - | - | **<0.001** |
| **Male PDET** | |  |  |  |  |  |  |
| Intercept | |  |  | 9.86 | 3.30 | 3.37, 16.35 | 0.003 |
| Cycle | Baseline (T0) | 6.6±3.8 | 7.0±3.8 | Reference | | | |
|  | 2 cycle (T1) | 6.5±3.9 | 6.8±3.9 | -0.24 | 0.34 | -0.90, 0.43 | 0.482 |
|  | 4 cycle (T2) | 6.3±4.3 | 7.1±3.3 | 0.14 | 0.36 | -0.57, 0.85 | 0.701 |
| Group | |  |  | -0.36 | 0.48 | -1.30, 0.58 | 0.203 |
| Group*Cycle | |  |  |  |  |  | 0.471 |
| Note:Values in bold are P < 0.05. ^#^: T2 vs. T0, *p*<0.001; Abbreviations: SE, standard error; CI, confidence interval; PSS, Perceived Stress Scale;FSFI, Female Sexual Function Index; IIEF, International Index of Erectile Function; PDET,Premature Ejaculation Diagnostic Tool. | | | | | | | |

| **Table S2** Cox regression analysis of time-to-pregnancy between groups (N = 254) | | | | | | | |
| --- | --- | --- | --- | --- | --- | --- | --- |
|  | Total | Pregnancy | Univariate analysis | |  | Multivariate analysis | |
|  |  |  | HR (95%CI) | *P* |  | HR (95%CI) | *P* |
| Ultrasound group | 127 | 51 | 2.106  (1.321, 3.359) | 0.002 |  | 2.120  (1.323, 3.394) | 0.002 |
| Test group | 127 | 27 |  |  |  |  |  |
| Abbreviations: HR, h**azard ratio**; CI, confidence interval. | | | | | | | |
